# Supplementary material for: Discovery of Novel Coumarin Derivatives as Potential Dual Inhibitors against α-Glucosidase and α-Amylase for the Management of Post-Prandial Hyperglycemia via Molecular Modelling Approaches
Source: Molecules. 2022 Jun 17;27(12):3888. doi: 10.3390/molecules27123888 (PMC9227442; doi:10.3390/molecules27123888)
Supplement: Supplementary file 1 [file molecules-27-03888-s001.zip › molecules-1749470-supplementary.pdf]

Supplementary Materials

Table S1. Virtual screening of coumarin derivatives against α-glucosidase (PDB ID: 3A4A) and α-amylase (PDB ID: 2QV4).

Coumarin Derivative	Binding Affinity (kcal/mol)		Total No of Intermolecular Interactions		Total No of Hydrogen Bonds	
	α-glucosidase	α-amylase	α-glucosidase	α-amylase	α-glucosidase	α-amylase
58	−11.9	−11.1	16	16	5	2
78	−11.8	−10.7	18	14	6	2
68	−11.8	−10.6	16	17	6	1
18	−11.8	−11.3	10	16	2	1
28	−11.7	−10.7	11	20	4	2
38	−11.7	−10.8	10	17	2	3
59	−11.6	−11.3	14	16	4	3
8	−11.6	−11.2	11	15	2	2
48	−11.5	−11.1	11	14	2	1
29	−11.4	−10.7	14	13	5	1
11	−11.4	−11	13	12	2	1
19	−11.4	−11.4	11	16	2	1
49	−11.3	−11.3	16	17	3	2
47	−11.3	−11.1	14	17	4	3
67	−11.3	−10.9	14	16	5	2
10	−11.3	−11.2	13	13	4	1
27	−11.3	−10.9	13	13	3	1
69	−11.3	−11	12	14	3	1
62	−11.2	−10.7	14	11	4	1
74	−11.2	−10.7	14	13	4	3
77	−11.2	−10.5	14	14	3	3
79	−11.2	−10.5	14	15	3	1
22	−11.2	−10.8	13	11	5	1
64	−11.2	−10.7	13	11	4	2
24	−11.2	−10.5	11	17	2	1
1	−11.2	−11.3	9	14	1	1
32	−11.1	−11	16	11	6	1
33	−11.1	−11	16	12	5	1
80	−11.1	−10.8	16	15	5	2
70	−11.1	−10.9	15	14	4	1
34	−11.1	−11	14	9	6	1
39	−11.1	−10.7	14	14	2	3
72	−11.1	−10.6	13	13	3	2
76	−11.1	−10.2	13	18	5	4
26	−11.1	−10.4	12	13	4	4
41	−11.1	−10.9	11	15	2	2
9	−11.1	−10.7	11	9	2	1
30	−11.1	−11	10	14	3	1
2	−11.1	−10.9	10	10	4	1
42	−11.1	−11	9	10	4	3
14	−11.1	−10.6	9	10	1	–
66	−11	−10.4	17	11	8	2
23	−11	−10.7	15	11	2	2
71	−11	−11	12	14	4	1

3	−11	−10.9	12	10	4	1
31	−11	−10.9	12	13	3	1
21	−11	−11	11	13	5	1
61	−11	−10.9	11	15	4	1
4	−11	−10.6	11	12	4	–
36	−11	−10.4	11	14	4	5
16	−11	−10.9	10	11	2	1
44	−11	−10.8	9	11	3	2
25	−10.9	−10.3	16	11	4	3
20	−10.9	−10.7	15	11	2	2
13	−10.9	−11.2	13	12	1	1
43	−10.9	−10.8	13	10	4	–
12	−10.9	−11.2	12	11	1	1
52	−10.9	−10.8	12	11	1	1
7	−10.9	−10.4	12	13	1	4
57	−10.9	−11	11	16	1	3
54	−10.9	−10.8	11	11	1	1
40	−10.9	−10.7	11	11	3	2
5	−10.9	−10.6	11	10	4	2
63	−10.9	−10.6	11	11	3	1
46	−10.9	−10.5	9	10	3	1
35	−10.8	−10.7	17	12	7	1
15	−10.8	−10.9	14	13	3	1
17	−10.8	−11.3	13	17	1	1
45	−10.8	−10.6	12	12	5	3
51	−10.8	−11.1	11	15	1	1
60	−10.8	−10.8	11	16	1	3
65	−10.8	−10.3	11	14	3	3
50	−10.8	−11.1	9	17	1	3
75	−10.7	−10.3	15	12	5	1
37	−10.7	−10.8	13	15	1	3
53	−10.7	−10.7	10	12	1	1
6	−10.7	−10.8	8	15	3	2
73	−10.6	−10.5	13	11	5	3
56	−10.6	−10.6	12	10	2	–
55	−10.5	−10.5	13	12	3	1
Acarbose	−7.9	−7.7	5	3	5	3

Table S2. Virtual screening of selected coumarin derivatives from pharmacophore studies for α-glucosidase.

Coumarin Derivative	Name	Binding Affinity (kcal/mol)	Total No of Intermolecular Interactions	Total No of Hydrogen Bonds
106	ZINC00629141	−10.8	9	2
42	ZINC21727023	−10.7	10	2
10	ZINC03144454	−10.6	12	1
103	ZINC15108551	−10.6	9	3
29	ZINC40949448	−10.5	14	5
105	ZINC63569231	−10.5	8	1
27	ZINC02789268	−10.4	9	2
66	ZINC02089876	−10.4	13	3
93	ZINC08397526	−10.4	10	4
2	ZINC03619802	−10.3	8	3
6	ZINC02151788	−10.3	15	4
83	ZINC09402980	−10.3	11	4
15	ZINC06767580	−10.2	13	4
31	ZINC16653680	−10.2	10	1
40	ZINC27571726	−10.2	9	4
54	ZINC38706202	−10.2	8	3
101	ZINC04871886	−10.2	10	1
11	ZINC41013606	−10.1	12	3
28	ZINC40949454	−10.1	12	4
49	ZINC09086839	−10.1	7	2
8	ZINC02768634	−10	12	3
86	ZINC01953161	−10	10	3
12	ZINC01953169	−9.9	12	2
34	ZINC41018460	−9.9	10	2
3	ZINC02789179	−9.8	15	3
26	ZINC02995395	−9.8	14	5
36	ZINC02794848	−9.7	10	2
57	ZINC41018448	−9.7	8	5
74	ZINC02103343	−9.7	11	4
85	ZINC40939457	−9.7	7	4
13	ZINC01953165	−9.6	10	2
37	ZINC02789197	−9.6	10	4
38	ZINC41018451	−9.6	11	5
39	ZINC01791774	−9.6	13	4
47	ZINC40949444	−9.6	11	4
48	ZINC02768466	−9.6	13	3
53	ZINC40928958	−9.6	10	2
21	ZINC01953167	−9.5	11	2
22	ZINC01979058	−9.5	9	2
64	ZINC40949442	−9.5	14	5
69	ZINC14245419	−9.5	6	1
71	ZINC00673528	−9.5	14	4
94	ZINC13892994	−9.5	9	3
102	ZINC04369253	−9.5	10	2
107	ZINC31991150	−9.5	12	4
18	ZINC02773952	−9.4	11	4
44	ZINC40949446	−9.4	12	5
56	ZINC40949450	−9.4	8	3
70	ZINC41013597	−9.4	14	3
72	ZINC02789445	−9.4	11	5
73	ZINC01911823	−9.4	10	2
76	ZINC00680045	−9.4	11	4
79	ZINC01783162	−9.4	5	1

90	ZINC00689025	−9.4	6	2
14	ZINC40949452	−9.3	11	5
17	ZINC02773953	−9.3	12	4
19	ZINC02768649	−9.3	11	3
23	ZINC01819786	−9.3	11	4
41	ZINC02768552	−9.3	10	3
82	ZINC02103025	−9.3	10	2
4	ZINC02789178	−9.2	14	3
35	ZINC02103711	−9.2	12	3
58	ZINC01783164	−9.2	10	1
75	ZINC02659617	−9.2	6	2
77	ZINC40949350	−9.2	8	2
78	ZINC01953157	−9.2	11	5
89	ZINC01953156	−9.2	11	3
95	ZINC00689171	−9.2	13	4
96	ZINC00731973	−9.2	8	3
99	ZINC41018437	−9.2	10	3
24	ZINC40939459	−9.1	11	3
25	ZINC02794859	−9.1	14	4
46	ZINC02865597	−9.1	13	3
60	ZINC02865598	−9.1	7	4
67	ZINC32526568	−9.1	11	4
88	ZINC01953155	−9.1	11	2
100	ZINC06720126	−9.1	9	1
43	ZINC01953163	−9	11	5
45	ZINC01979047	−9	10	3
55	ZINC01979046	−9	11	3
61	ZINC02103468	−9	13	3
98	ZINC02763941	−9	10	3
1	ZINC08454361	−8.9	10	2
51	ZINC14052601	−8.9	9	3
62	ZINC01953159	−8.9	14	7
63	ZINC01112858	−8.8	8	3
65	ZINC01979048	−8.8	12	4
92	ZINC08387404	−8.8	10	2
97	ZINC07996269	−8.8	9	3
33	ZINC28540235	−8.7	13	4
59	ZINC02763876	−8.7	11	3
80	ZINC02759723	−8.7	7	1
81	ZINC01979045	−8.7	8	2
87	ZINC40939461	−8.7	11	3
104	ZINC12865181	−8.7	8	3
50	ZINC32526616	−8.5	11	5
84	ZINC78814674	−8.5	14	1
30	ZINC00659412	−8.4	13	3
7	ZINC02768133	−8.3	10	3

Table S3. Virtual screening of selected coumarin derivatives from pharmacophore studies for α-amylase.

Coumarin Derivative	Name	Binding Affinity (kcal/mol)	Total No of Intermolecular Interactions	Total No of Hydrogen Bonds
15	ZINC03185211	−9.4	6	−
20	ZINC08721887_1	−9.4	6	−
47	ZINC21884078_1	−9.4	6	−
29	ZINC09634265	−9.3	9	−
30	ZINC13120435_1	−9.3	6	1
40	ZINC15799331_1	−9.3	6	−
3	ZINC01850100	−9.2	9	1
12	ZINC03144061_5_2	−9.2	8	−
16	ZINC05350534_1_1	−9.2	7	1
25	ZINC08721887_6	−9.2	8	−
26	ZINC08721887_7	−9.2	8	1
27	ZINC08721887_8	−9.2	8	−
34	ZINC13120435_7	−9.2	8	−
35	ZINC13120435_8	−9.2	7	−
37	ZINC13120435_10	−9.2	8	−
38	ZINC13120435_11	−9.2	8	−
45	ZINC15799331_6	−9.2	9	1
54	ZINC13496808	−9.2	18	2
2	ZINC01837889	−9.1	8	−
4	ZINC02162580	−9.1	8	1
13	ZINC03144061_6	−9.1	7	−
14	ZINC03144065	−9.1	8	−
19	ZINC08386500	−9.1	8	1
33	ZINC13120435_6	−9.1	8	−
36	ZINC13120435_9	−9.1	8	1
39	ZINC13120435_13	−9.1	8	−
1	ZINC00882659	−8.7	17	4
53	ZINC40949448	−8.2	19	1
56	Acarbose	−7.7	2	2
